# Supplementary material for: Fano resonant optical coatings platform for full gamut and high purity structural colors
Source: Nat Commun. 2023 Jul 5;14:3960. doi: 10.1038/s41467-023-39602-2 (PMC10322987; doi:10.1038/s41467-023-39602-2)
Supplement: Supplementary file 1 — Supplementary Information [file 41467_2023_39602_MOESM1_ESM.docx]

**Supplementary Information**

**Fano Resonant Optical coatings platform for Full Gamut and High Purity Structural Colors**

Mohamed ElKabbash^1,2, †, *,^ Nathaniel Hoffman^3, †^, Andrew R. Lininger^3, †^, Sohail A. Jalil^1^, Theodore Letsou^3^, Michael Hinczewski^3, *^, Giuseppe Strangi^3,4 *^, and Chunlei Guo^1, *^

1. The Institute of Optics, University of Rochester, Rochester, NY 14627, USA.
2. Current address: College of Optical Sciences, University of Arizona, Tucson, AZ, 85721, USA.
3. Department of Physics, Case Western Reserve University, 10600 Euclid Avenue, Cleveland, Ohio 44106, USA.
4. CNR-NANOTEC and the Department of Physics University of Calabria, Rende (Italy).

* Corresponding emails: [melkabbash@arizona.edu](mailto:melkabbash@arizona.edu) (M.E.), [mxh605@case.edu](mailto:mxh605@case.edu) (M.H.),

[gxs284@case.edu](mailto:gxs284@case.edu) (G.S.), [guo@optics.rochester.edu](mailto:guo@optics.rochester.edu) (C.G.).

†These authors contributed equally.

The simulation and optimization code is freely available on Git-Hub in this link: [*https://github.com/hincz-lab/structural_color_FROCs*](https://github.com/hincz-lab/structural_color_FROCs)
(DOI: 10.5281/zenodo.78934)


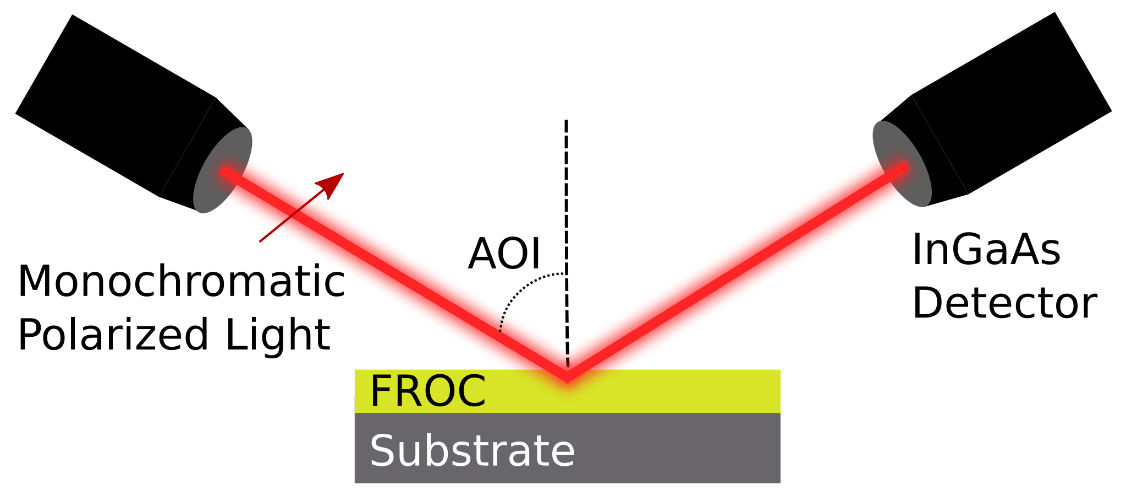


**Figure S1| Experimental setup** The reflectance measurements were done using a variable-angle high-resolution spectroscopic ellipsometer.


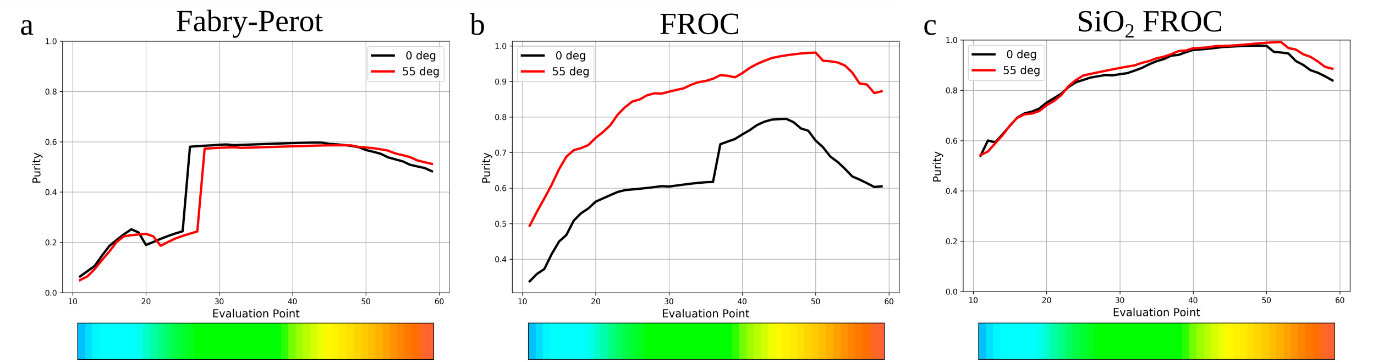


**Figure S2|** **Color purity of FROCs** The color purity of (a) Fabry-Perot cavities, (b) FROCs, and (c) silica capped FROCs (SiO_2_ FROCs) at 0 deg and 55 deg angle of incidence. Purity levels > 99% can be achieved for SiO_2_ FROCs over a wide range of angles. ‘Evaluation Point’ refers to the wavelength at which the purity is evaluated (color shown below).


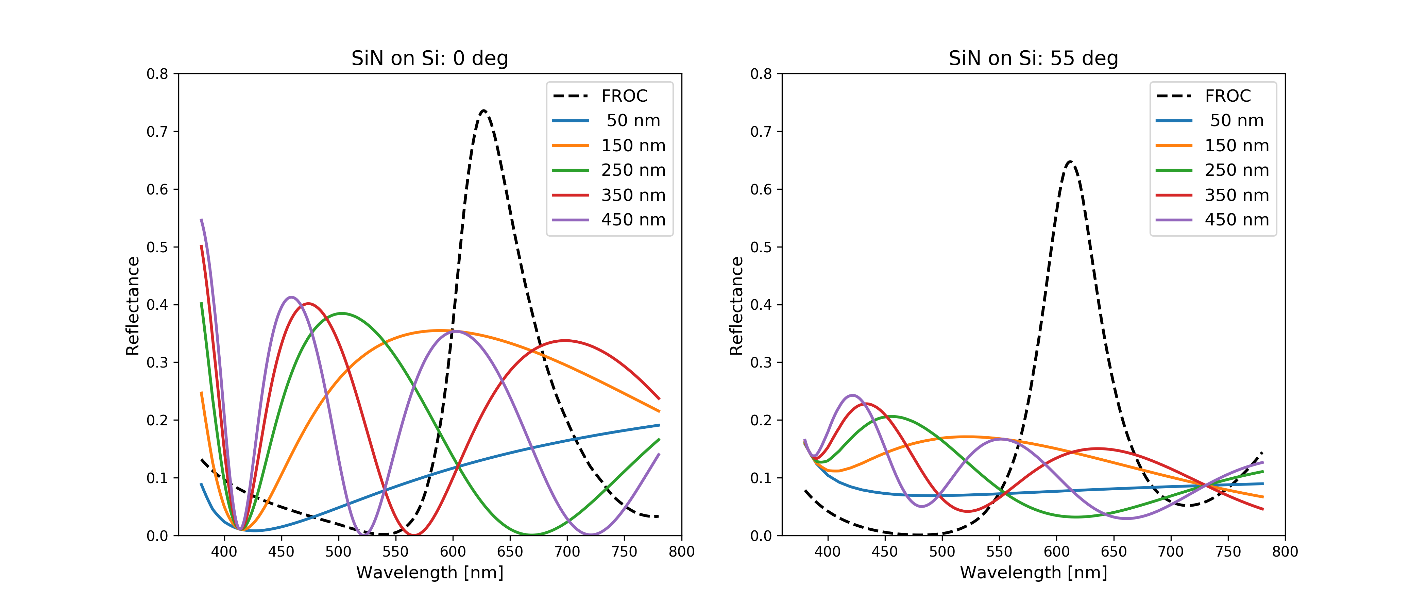


**Figure S3| SiN structural color comparison** Comparison of the reflectance for SiN on a Si substrate vs. FROCs. The SiN/Si system is simulated for a range of thicknesses. Although the Fabry-Perot resonance results in a reflection peak, the colors suffer from low brightness.


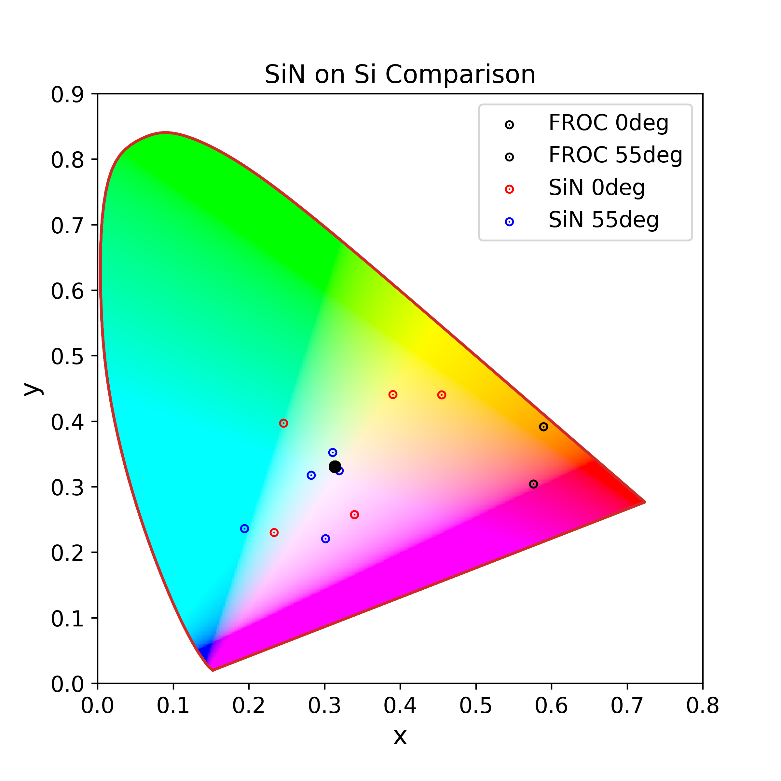


**Figure S4|** **SiN CIE gamut** CIE color space representation for the reflectance spectra presented in **Figure S3**. The color purity of the FROC is significantly larger than that of SiN/Si system.


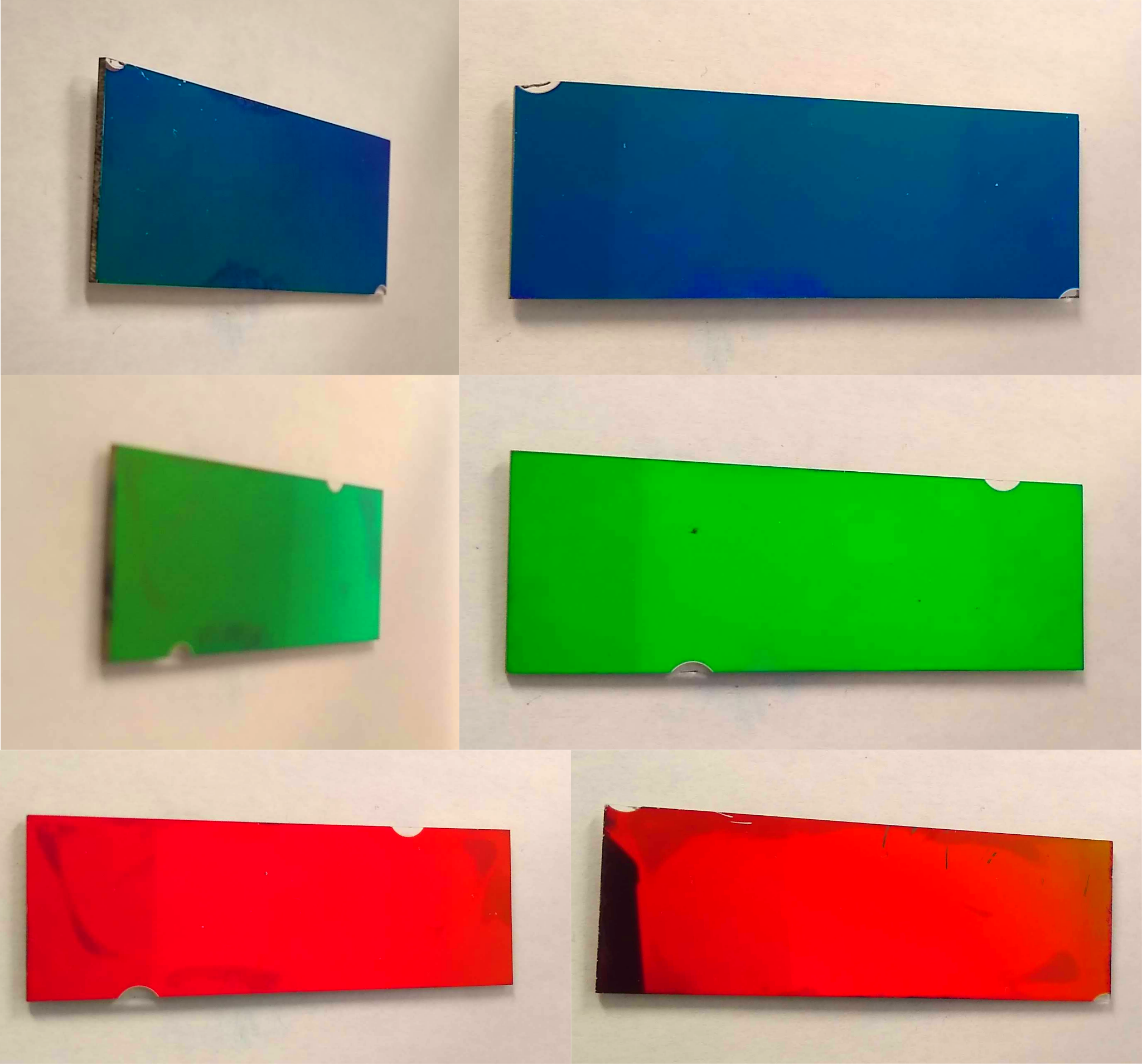


**Figure S5|** **Silica capped FROC photographs** Photographs of experimentally fabricated silica capped FROC samples illustrating blue (top), green (middle), and red (bottom) structural color resonances. The samples are illuminated with a F11 type illuminant spectrum.


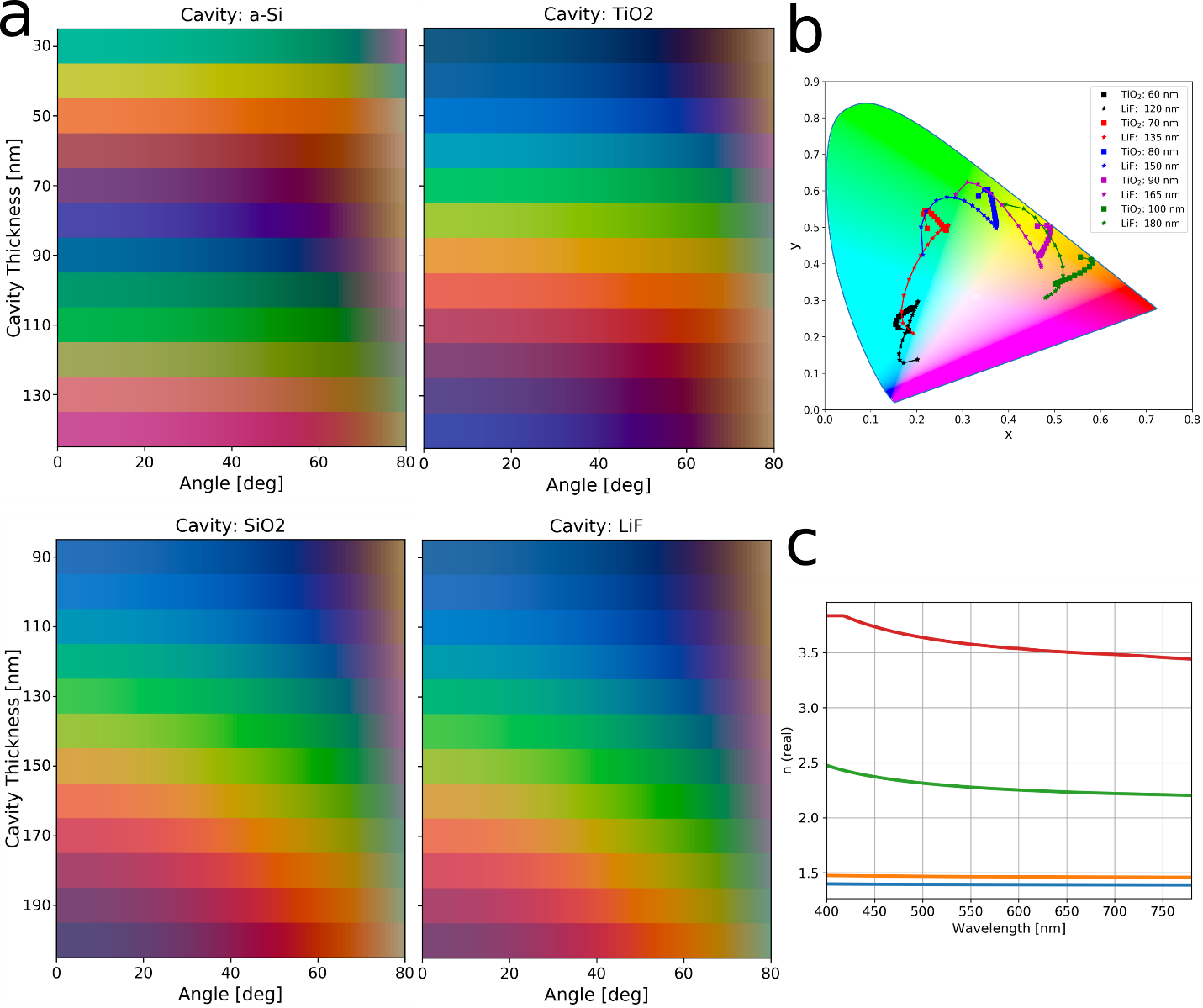


**Figure S6| Iridescence of structural color FROCs** Iridescence properties of FROCs with a range of cavity materials at different refractive indices. **(a)** Iridescence plots of FROCs with different cavity materials (a-Si, TiO_2_, SiO_2_, and LiF) and with a range of cavity thicknesses. The iridescence properties can be controlled by changing the material in the cavity. For high index materials, the color is relatively consistent for a wide range of incidence angles. **(b)** Gamut showing the color changes for TiO_2_ (squares) and LiF (stars) cavity FROCs varying the angle of incidence from 0 deg to 65 deg at several cavity thicknesses. **(c)** Real refractive indices for the cavity materials in (**c**).


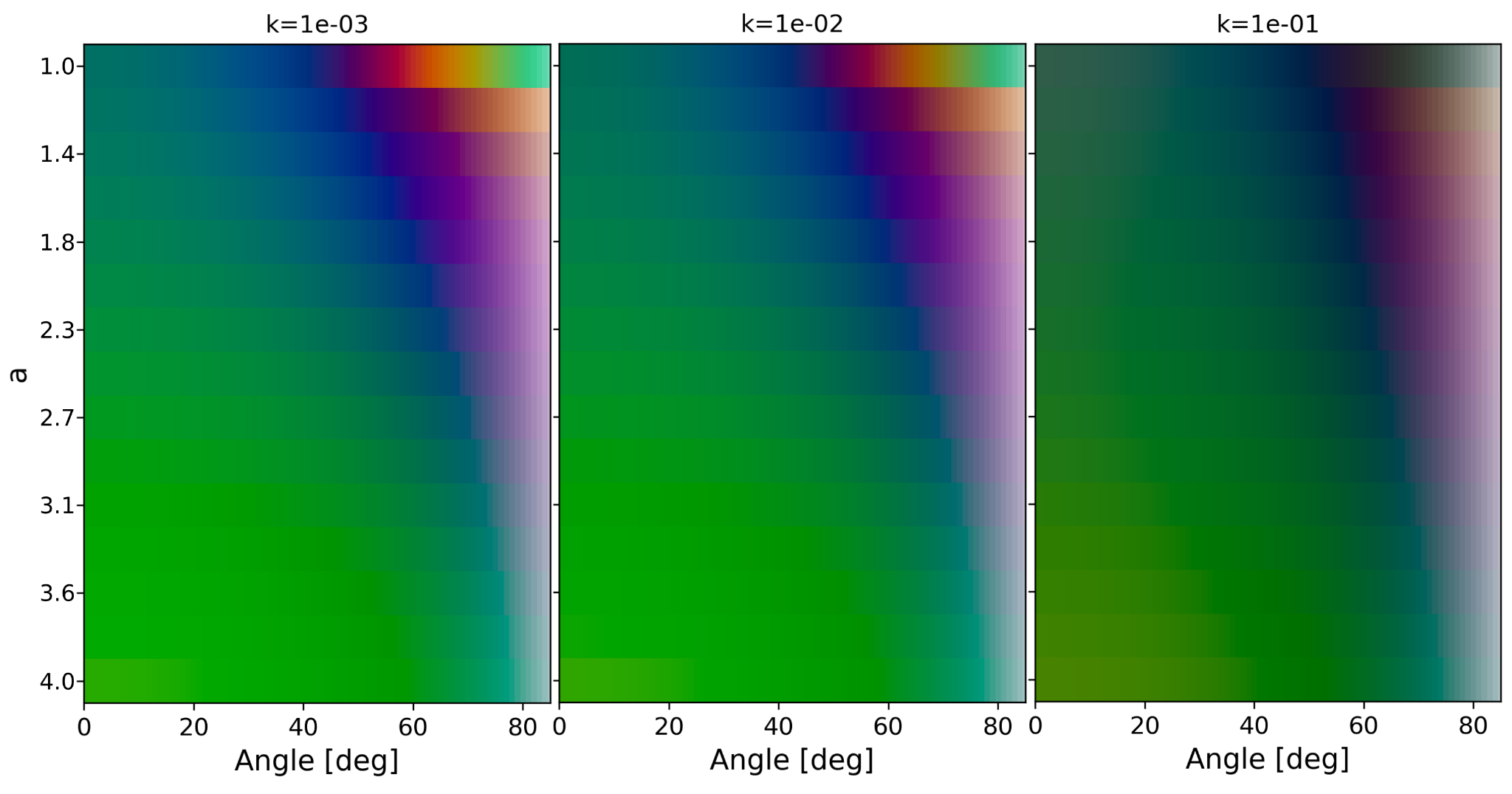


**Figure S7|** **Iridescence from Cauchy dispersions** Iridescence properties of FROCs utilizing cavity materials with a Cauchy dispersion and constant extinction of 1E-3 (left), 1E-2 (center), and 1E-1 (right). The results are qualitatively similar to the simulated iridescence with dispersionless cavity materials.


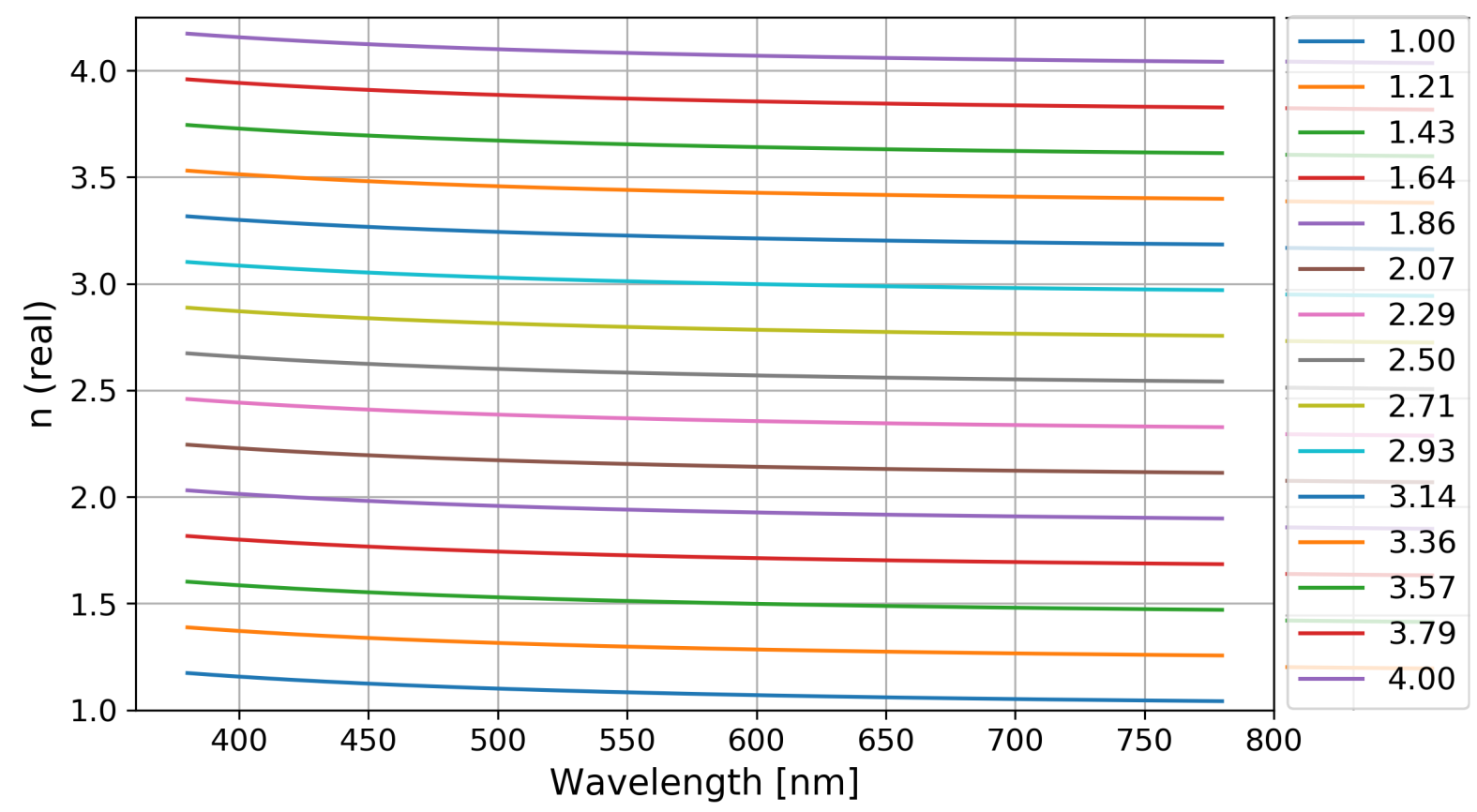


**Figure S8| Cauchy dispersions** Cauchy dispersions considered in **Figure S7**, for various values of a, with b=0.025. These dispersions represent a good approximation of many lossless dielectric materials in the visible range.


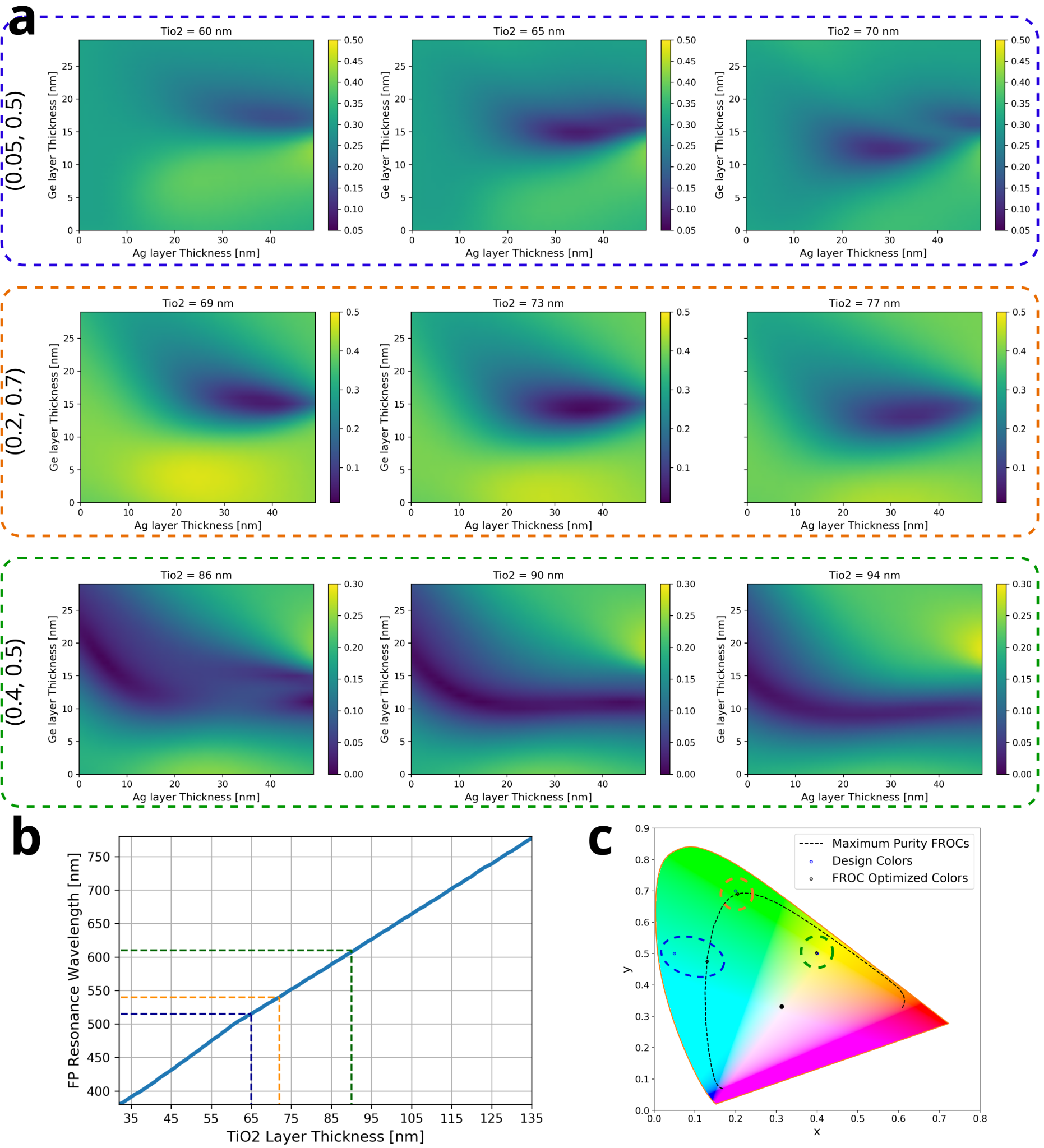


**Figure S9| Optimization process for structural color FROCs** Illustration of the optimization protocol for structural coloring using FROCs. **(a)** Three examples are presented for FROC structural coloring for blue, orange, and green, from top to bottom to optimize maximum purity levels. The RMSE x-y distance space is shown for different values of Ge (y), Ag (x), and TiO2 (plot) layer thicknesses. In each case the RMSE space is relatively uncomplicated with a visible global minimum. **(b)** The Fabry-Perot resonance wavelength changes linearly with TiO2 layer (cavity) thickness for a MIM cavity. Peak wavelength for the FROC can be tuned by controlling the TiO2 layer thickness. **(c)** CIE color space showing the target color response and optimized FROC system for the three cases presented in (a). The optimization protocol accurately predicts structures up to the maximum purity level.


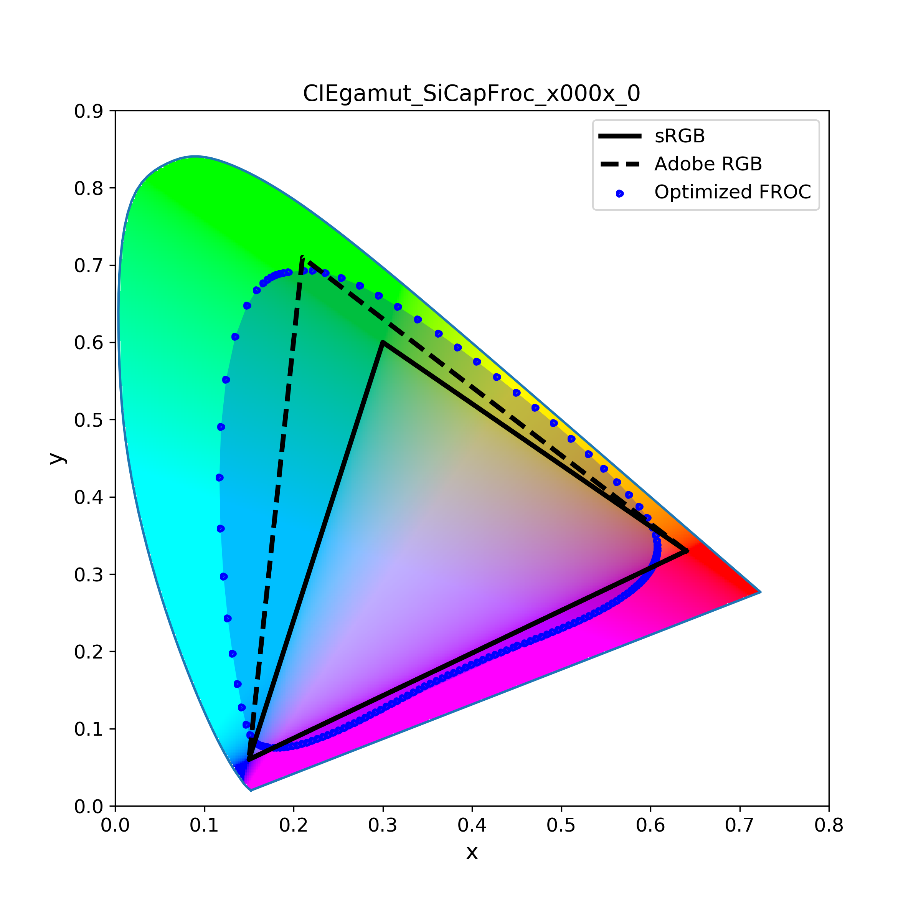


**Figure S10| sRGB and Adobe RGB color space comparisons** CIE 1931 chromaticity diagram comparing optimized Silica-capped FROCs with sRGB color space and Adobe RGB color space. The total area covered by the Silica-capped FROCs is 131.6% and 177.5% compared to the Adobe RGB and sRGB color subspaces, respectively.


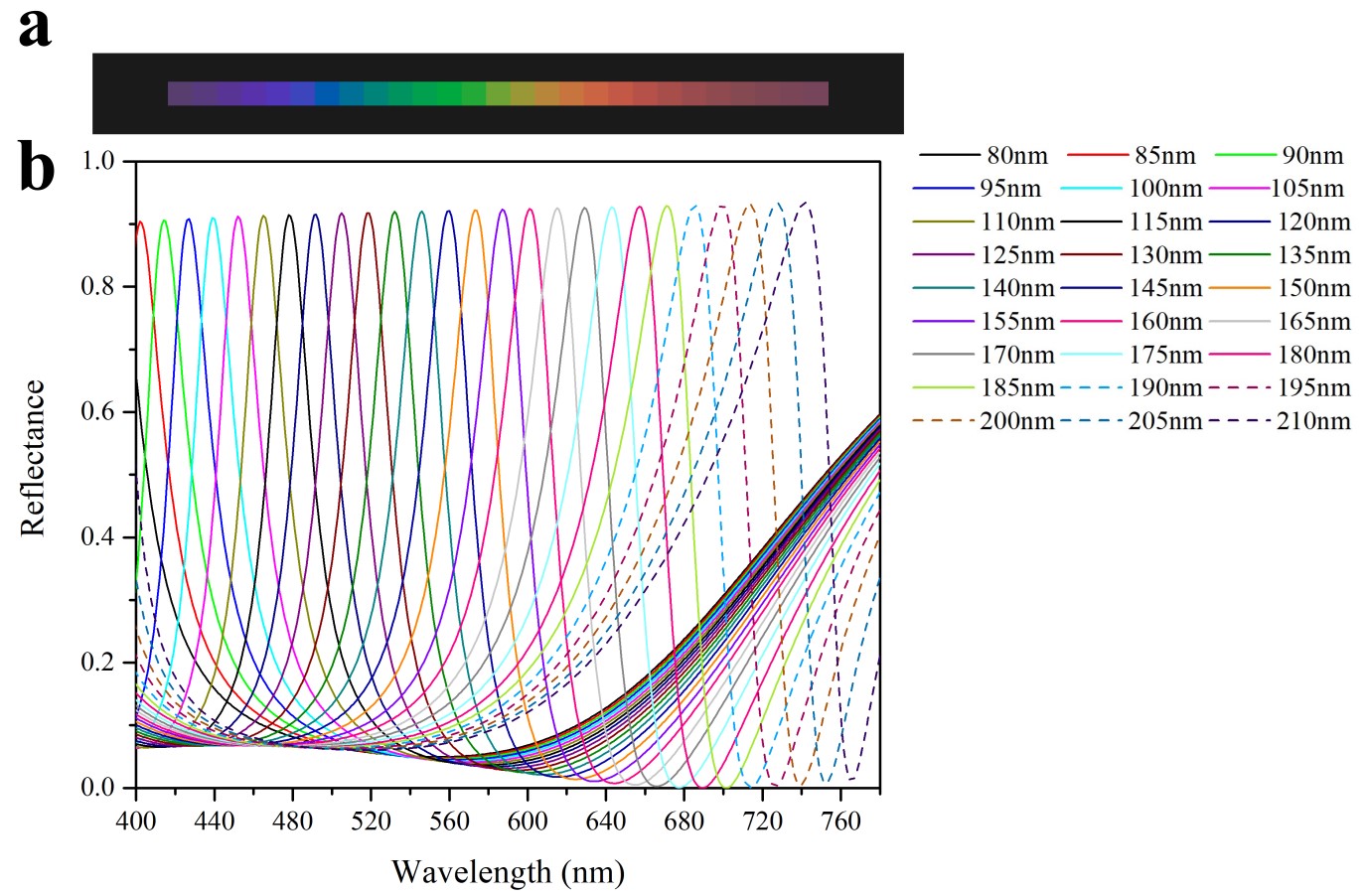


**Figure S11| Brightness of FROCs (a)** The calculated colors corresponding to FROCs cavity by varying the dielectric (SiO_2_) thickness from 80 nm to 210 nm. The narrow reflection lines shown in **(b)** lead to high purity colors.


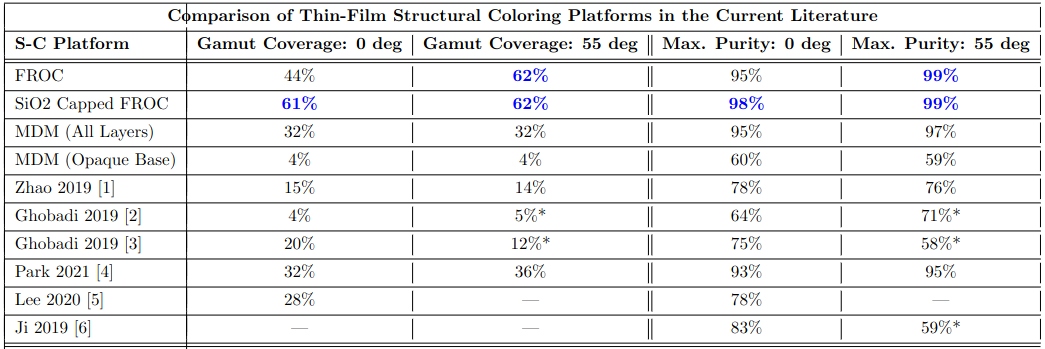
** Angle of incidence is 60 deg*

**Table S1| Comparison of Thin-Film Structural Coloring Platforms** Total gamut coverage and maximum purity of FROCs compared with thin-film structural coloring platforms for 0 deg and 55 deg angles of incidence. The best performing system in each category is highlighted in blue.

**Supplementary Note 1:**

Mathematically, the angle dependence of the FROC resonant reflection mode depends entirely on the properties of the MDM cavity. The wavelength of the reflectance peak $\lambda_{max}$ depends on the incident angle and is given by:

|  | $\frac{1}{\lambda_{max}\left( \theta\right)}\frac{d\lambda_{max}\left( \theta\right)}{d\theta} \sim H\left( \lambda_{max}\left( \theta\right),\theta,n_{d} \right)\frac{coscos\theta sinsin\theta}{n_{d}^{2}-\theta},$ |  |
| --- | --- | --- |

where $H\left( \lambda_{max}\left( \theta\right),\theta,n_{d} \right)$ is a dimensionless function that depends on solely on $\theta$ through $\lambda_{max}$. As $n_{d}$ increases to values >>1, the above expression decreases as $n_{d}^{-2}$. Accordingly, the iridescence of FROCs can be mitigated significantly by using a high index dielectric.

**Supplementary Note 2:**

The simplicity of the FROC structural coloring platform leads to a relatively straightforward optimization problem in most cases. We adopted the root mean square error optimization process (RMSE). **Figure S9** shows the RMSE optimization process for blue, orange and green. In all the cases shown, the RMSE x-y distance space is simple and has a smooth global minimum. Furthermore, the reflectance peak location depends mainly on the dielectric cavity thickness, which determines the Fabry-Perot cavity resonance wavelength (**Figure S9b**). Accordingly, the RMSE space for FROCs is simple and enjoys low dimensional correlation, meaning that each layer in the structure has a discernably different effect upon the optical response. These factors justify the adopted straightforward optimization protocol. FROC structures were optimized to find the gamut coverage by performing a bounded 50 (or 60 in some cases) point random global least-squares (Trust Region Reflective algorithm) fit for the layer thicknesses. In the case of the silica capping layer, thickness optimization of the silica cap was performed by hand. Based on the considerations above, we believe this protocol is sufficient to fully cover the design space and that more sophisticated optimization techniques are unnecessary for this problem. The optimization code is freely available on Git-Hub using this link“ <https://github.com/hincz-lab/structural_color_FROCs> “ (DOI: 10.5281/zenodo.78934)

**Supplementary References:**

[1] Zhao, J. *et al.* Defining Deep‐Subwavelength‐Resolution, Wide‐Color‐Gamut, and Large‐Viewing‐Angle Flexible Subtractive Colors with an Ultrathin Asymmetric Fabry–Perot Lossy Cavity. *Advanced Optical Materials* **7**, 1900646 (2019).

[2] Ghobadi, A., Hajian, H., Gokbayrak, M., Butun, B. & Ozbay, E. Bismuth-based metamaterials: from narrowband reflective color filter to extremely broadband near perfect absorber. *Nanophotonics* **8**, 823-832 (2019).

[3] Ghobadi, A., Hajian, H., Soydan, M. C., Butun, B. & Ozbay, E. Lithography-free planar band-pass reflective color filter using a series connection of cavities. *Scientific Reports* **9**, 1-11 (2019)

[4] Park, C.-S. & Lee, S.-S. Vivid coloration and broadband perfect absorption based on asymmetric Fabry–Pérot nanocavities incorporating platinum. *ACS Applied Nano Materials* **4**, 4216-4225 (2021).

[5] Lee, Junho, Jaeyong Kim, and Myeongkyu Lee. High-purity reflective color filters based on thin film cavities embedded with an ultrathin Ge2Sb2Te5 absorption layer. *Nanoscale Advances* **2,** 10, 4930-4937 (2020).

[6] Ji, Chengang, Kyu-Tae Lee, and L. Jay Guo. High-color-purity, angle-invariant, and bidirectional structural colors based on higher-order resonances. *Optics Letters* **44**, 1, 86-89 (2019).
